# 2024 Acknowledgment of *mBio* Invited Editors

**DOI:** 10.1128/mbio.03933-24

**Published:** 2025-02-05

**Authors:** Arturo Casadevall

**Affiliations:** 1 Johns Hopkins Bloomberg School of Public Health, Baltimore, Maryland, USA

## EDITORIAL

On behalf of the editors of *mBio*, I gratefully acknowledge the following individuals, who served as invited editors for the journal from November 2023 to November 2024. While not members of the board of editors, invited editors play an important role in the review process. Invited editors are experts in their fields of research who contribute an additional level of quality to the review process. An editor may assign a paper to an invited editor when they would like to have an additional expert opinion of the reviews or when the subject area falls outside the editor’s primary area of expertise.

Paul Ahlquist

Christopher Aiken

Bree Aldridge

Holly Scott Algood

Francis Alonzo

Germán Andrés

Fernando Anhe

Josefa Antón

Manuel Aranda

Elizabeth Arnold

Luciana Arruda

Frank Aylward

Adam Bailey

Fernando Baquero

Antonio Barragan

Christopher Basler

Himanshu Batra

Anthony Baughn

Patrik Bavoil

Richard Bennett

Diane Bolton

Daniel Bond

Erhard Bremer

Igor Brodsky

Cara Brook

Christopher Brooke

Ilana Camargo

Scott Canfield

Eric Cascales

Louise Cerdeira

Kartik Chandran

Bobby Cherayil

Matthew Child

Ewa Chrostek

Camila Coelho

Jeffrey Coleman

Laurie Comstock

John Connor

James Cotton

Lynda Coughlan

Laura Cox

Ajai Dandekar

Lawrence David

Ian Davis

Maurizio Del Poeta

Nicole De Nisco

Terence Dermody

Katja Dierking

Lars Dietrich

Daniel DiMaio

George Dimopulos

Dirk Dittmer

Michał Dmowski

Maria Doligalska

Breck Duerkop

Gregory Ebel

Patrick Eichenberger

Ying Fang

Herman Favoreel

Mario Feldman

Paul Fey

Andrew Firth

Ana Flores-Mireles

Annette Fox

Ashwanth Francis

Megan Freeman

Matthew Frieman

Marco Galardini

Nicole Gerardo

Benjamin Gewurz

Stephen Giovannoni

Alan Goodman

Friedrich Götz

Elizabeth Grice

Marc-Jan Gubbels

David Guttman

Ted Hackstadt

Matthias Hahn

Steven Harris

Alistair Harrison

Stanley Hazen

David Heinrichs

Sophie Helaine

Bobbi Helgason

David Hendrixson

David Hibbett

Russell Hill

Fred Homa

Suchitra Hourigan

Jianming Hu

Victor Huber

Bruce Hungate

David Hunstad

Christopher Hunter

Jean-Luc Imler

Federico Iovino

Swati Jain

Janet Jansson

Theodore Jardetzky

Dong-Yan Jin

Kirsten Jung

Regine Kahmann

Thomas Kehl-Fie

Kylene Kehn-Hall

Alyson Kelvin

Tammy Kielian

Samantha Jane King

Natalia Kirienko

Karl Klose

Denis Kolbasov

Jay Kolls

Anna Konovalova

Jeroen Koomen

Richard Kuhn

Smita Kulkarni

Salome Landmann

Karl Lechtreck

Jose Lemos

John Leong

Daniel Lessner

Kim Lewis

Melody Li

Andrew Limper

Shan-Lu Liu

Per Ljungdahl

Robert Luallen

Carolyn Machamer

Anthony Maresso

Pablo Marquet

David Matthews

Vineet Menachery

William Metcalf

Edward Mocarski

Trudy Morrison

Rafal Mostowy

Poonam Mudgil

Karl Münger

William Navarre

Irene Newton

Caroline Ng

Evgeny Nudler

Catherine O'Connell

Michinaga Ogawa

Teresa O'Meara

George O'Toole

Michael Otto

Sivasankar Palaniappan

Søren Paludan

Daniel Paredes-Sabja

John Parker

Teresa Pawlowska

Shelley Payne

Andrew Pekosz

Rushika Perera

Alexandre Persat

Sebastien Pfeffer

Paul Planet

Abani Pradan

John Rawls

Felix Rey

Juergen Richt

Jason Rosch

David Rosen

Benjamin Ross

David Sacks

Peter Sarnow

William Schneider

Robert Schooley

Leon Schulte

Einat Segev

Stephanie Seifert

Bert Semler

Nathan M. Sherer

Steven Sinkins

Nicolas Sluis-Cremer

Alison Smith

Lotte Søgaard-Andersen

Paul Spearman

Alfred Spormann

Kenneth Stapleford

Spyridon Stavrou

Helene Strick-Marchand

Wesley Sundquist

Staffan Svärd

Malak Tfaily

Niraj Tolia

Liang Tong

Emily Troemel

Carolina Tropini

B. Gillian Turgeon

Julia van Kessel

Andres Vazquez-Torres

Joseph Wade

Jennifer Walker

Aiming Wang

Chengshu Wang

Jin-Town Wang

Matthew Wargo

Richard Webby

James Weedon

James Weger-Lucarelli

David Weiss

Matthew Weitzman

Joel Wertheim

Angela Wilks

David Wilson

Emma Wilson

Alan Wolfe

Guojun Wu

Shuqi Xiao

Joanne Yew

María Zambrano

Chunfu Zheng

